# Observations on the Treatment of Pneumonia

**Published:** 1808-09

**Authors:** 


### *To the Editors of the Medical and Physical Journal.*

GENTLEMEN,

TRUSTING that the purport of your publication is to cherish every attempt that may tend in the least to the improvement and consequent advancement of medicine, I have ventured to solicit the insertion of the following observations, subjoined with a case; and though my experience may not entitle my sentiments to that respect and attention, that older disciples of Galen deservedly merit, yet it is hoped that my sincere wishes for the improvement of the art may tend to palliate my defects, and shield me from criticism. The subject on which I wish to offer some remarks, is the treatment of Pneumonia, a disease of some importance, and to which the inhabitants of this varying clime are extremely subject, owing most probably to the unsettled state of the atmosphere. I pretend to no new discovery, nor any novel application of an established remedy, I only wish to recommend to the notice of others, (who are more competent judges than myself,) a practice of which I have seen the beneficial effects repeatedly in this acute disease. The remote causes of pneumonia, in my opinion, are either cold applied to the body obstructing perspiration, and determining to the lungs, or else injury to the pneumonic organs from some external cause, viz. violent blows, strains, or any such violence as may produce inflammation; and these circumstances more particularly affect those persons of an inflammatory habit, and of a robust and vigorous constitution. But from whatever cause it may proceed, we may safely judge of the disease from concomitant symptoms, such as pyrexia, cough, dyspnœa, pain in some part of the thorax, increased frequency of pulse, &c. all which characterise the disease, so that it is almost impossible to be mistaken; but as the treatment of the disease, and not the definition of it, is the principal object I have in view, I shall enlarge no further in this particular, but advert to the treatment of the disorder. The principal remedy generally depended upon, has been the free, and, in some instances, almost unlimited use of the lancet. The reason for which I hope is better known to those who are greater friends to venesection in this disease, than I am at present. I can see no necessity for bleeding, except under certain circumstances; it undoubtedly may be of essential service in the commencement mencement of the complaint, when expectoration has not commenced, and the strength of the patient is not diminished by the attack; here you may, by the use of the lancet, remove inflammation, and perhaps stop the progress of the disease, before the symptoms of danger appear; likewise in robust and athletic constitutions, where the vis vitæ is undiminished and the circulation of blood rapid; in both these instances venesection stands pre-eminent over every other active remedy. It is a notorious fact, that pneumonia more commonly terminates in expectoration than not. Now, it has been alleged by some experienced practitioners, that you are not to neglect bleeding even after the fourth day, and that expectoration is not in the least to supersede it. Is this consonant with reason or experience? When expectoration has once commenced, will not bleeding copiously at the arm, and persisting in it till it almost produces syncope, as some writers have advised, tend to stop that expectoration, which nature has formed to carry off the disease, and materially weaken the vital energies of the constitution, and rob it of that support it necessarily requires, to bear up against the disease? Blisters I have witnessed of essential service in this malady; the modus operandi of which I cannot attempt to explain exactly, but I think they tend to lessen inflammation, and assist nature in an eminent degree; and if they were more generally adopted, their utility would consequently be more generally known; but I am confident of this, that in four cases out of five there is no necessity at all for bleeding, and that the majority of sufferers in this complaint would be more likely to recover, were the lancet less freely used. Perhaps the relation of the following short case may tend to illustrate the preceding remarks. A man, about 30, came to me for relief, attacked with this disorder in a most violent degree; respiration dreadfully laborious, so much so, that he almost felt the sense of suffocation, (with expectoration of yellow mucus) pulse 110; acute pain in the thorax, with heat and fever, &c. The man was not in good circumstances, and had delayed obtaining medical assistance for two days or more, which tended in some measure to aggravate the disorder. I immediately ordered him a *large blister*, to be applied to the chest, and an expectorating mixture with g. ammon and squills, &c. In the evening he was much better; could respire easier; the pain in the thorax not so great; pulse still high, though much abated; could only lay on his back, and now felt acute pain in the left side, for which I ordered

 him him another *large blister*, to be placed on the part in pain. Next day he was considerably better in every respect, but extremely low and feverish. He drank the decoct. hordei. compt. constantly for common drink, and I made the camphor julep the menstruum of his expectorating mixture. He followed up this plan for two or three days longer, till there was no symptom of inflammation left; I now gave him tonics, and he took gentle exercise, and in about ten days the man went about his usual employment. I shall now leave the preceding observations, and single case, out of many others, in which I have been witness of the beneficial effects of blisters, to the perusal of your intelligent readers, and shall feel myself obliged to them if I have advanced any thing that is palpably erroneous, to set me right, and close with one remark, that I think if I had bled in the above case till urgent symptoms had disappeared, in his state of debility and weakness, I should soon have brought him to his last home.

VERITAS.

